# The influence of socioeconomic deprivation on multimorbidity at different ages: a cross-sectional study

**DOI:** 10.3399/bjgp14X680545

**Published:** 2014-06-30

**Authors:** Gary McLean, Jane Gunn, Sally Wyke, Bruce Guthrie, Graham CM Watt, David N Blane, Stewart W Mercer

**Affiliations:** Institute of Health and Wellbeing, University of Glasgow, Glasgow, UK.; Primary Care Research Unit, University of Melbourne, Melbourne, Australia.; Institute of Health and Wellbeing, University of Glasgow, Glasgow, UK.; Quality, Safety and Informatics Research Group, University of Dundee, Dundee, UK.; Institute of Health and Wellbeing, University of Glasgow, Glasgow, UK.; Institute of Health and Wellbeing, University of Glasgow, Glasgow, UK.; Institute of Health and Wellbeing, University of Glasgow, Glasgow, UK.

**Keywords:** chronic disease, mental health, multimorbidity, primary health care, socioeconomic status

## Abstract

**Background:**

Multimorbidity occurs at a younger age in individuals in areas of high socioeconomic deprivation but little is known about the ‘typology’ of multimorbidity in different age groups and its association with socioeconomic status.

**Aim:**

To characterise multimorbidity type and most common conditions in a large nationally representative primary care dataset in terms of age and deprivation.

**Design and setting:**

Cross-sectional analysis of 1 272 685 adults in Scotland.

**Method:**

Multimorbidity type of participants (physical-only, mental-only, mixed physical, and mental) and most common conditions were analysed according to age and deprivation.

**Results:**

Multimorbidity increased with age, ranging from 8.1% in those aged 25–34 to 76.1% for those aged ≥75 years. Physical-only (56% of all multimorbidity) was the most common type of multimorbidity in those aged ≥55 years, and did not vary substantially with deprivation. Mental-only was uncommon (4% of all multimorbidity), whereas mixed physical and mental (40% of all multimorbidity) was the most common type of multimorbidity in those aged <55 years and was two- to threefold more common in the most deprived compared with the least deprived in most age groups. Ten conditions (seven physical and three mental) accounted for the top five most common conditions in people with multimorbidity in all age groups. Depression and pain featured in the top five conditions across all age groups. Deprivation was associated with a higher prevalence of depression, drugs misuse, anxiety, dyspepsia, pain, coronary heart disease, and diabetes in multimorbid patients at different ages.

**Conclusion:**

Mixed physical and mental multimorbidity is common across the life-span and is exacerbated by deprivation from early adulthood onwards.

## INTRODUCTION

Multimorbidity, defined here as the coexistence of two or more chronic conditions in the same individual, presents a challenge to patients, clinicians, healthcare systems, and researchers.^1^–^4^ Multimorbidity is associated with poor health outcomes, including higher mortality^5^,^6^ and lower quality of life,^7^ resulting in more complex healthcare needs^8^ and higher healthcare costs.^9^,^10^ Estimates of the prevalence of multimorbidity vary depending on the study population, the definition of multimorbidity used, and the use of different numbers and definitions of conditions.^11^,^12^ Regardless of how it is measured, multimorbidity is more common in older people,^13^,^14^ and in more deprived populations.^15^

To date, research on multimorbidity has mainly focused on older people.^16^–^19^ However, several studies have shown that multimorbidity is not just a problem of old age.^15^,^20^,^21^ Indeed, a recent large study found more people with multimorbidity aged <65 than ≥65 years.^15^ Higher rates of multimorbidity in younger age groups are particularly common in deprived areas.^15^,^22^ Studies that have examined multimorbidity across age groups have generally been small, included only a small number of conditions, or have been based on self-reporting.^16^–^18^

A life-course approach to understanding the aetiology of chronic diseases has been well documented for single physical conditions,^23^ and more recently for single mental conditions such as depression.^24^ A recent systematic review, however, found few prospective cohort studies specifically designed to investigate multimorbidity.^11^ Therefore, the aim of this study was to characterise the prevalence and type of multimorbid conditions by socioeconomic status across different age groups, using cross-sectional data from a large, nationally representative primary care dataset, to help inform future prospective studies.

## METHOD

The dataset was obtained from the Primary Care Clinical Informatics Unit at the University of Aberdeen, UK, and included copies of clinical data for 1 751 841 patients of all ages, permanently registered with 314 Scottish general practices, who were alive on 31 March 2007.^15^ The dataset consisted of complete copies of clinical data for all registered patients caring for about one-third of the Scottish population. Participating practices systematically used electronic medical records for registration of patients, morbidity recording, and prescriptions. The dataset has the same age and sex profile and a similar socioeconomic distribution to the Scottish population overall.^25^ The NHS National Research Ethics Service have previously approved the use of these anonymised data for research purposes and this analysis did not require independent review.^15^ Socioeconomic status was measured by the postcode-derived Carstairs score (grouped into tenths of the distribution), which is widely used for research purposes.^26^,^27^ Individuals were placed in deciles based on the range for the whole of Scotland.

**How this fits in**

Multimorbidity is more common and occurs at a younger age in individuals in areas of high socioeconomic deprivation, but the ‘typology’ of multimorbidity in different age and socioeconomic groups is not well characterised. Multimorbidity of physical conditions only is most common in those aged ≥55 years, and is not substantially related to socioeconomic status, whereas mixed physical and mental multimorbidity is most common in those aged <55 years and is two- to threefold more common in the most deprived compared with the least deprived. Ten conditions (seven physical and three mental) accounted for the top five most common conditions in those with multimorbidity in all age groups, with depression and pain featuring in the top five conditions in all age groups. Multimorbidity type thus varies substantially with age and deprivation, and understanding the illness trajectories of people with multimorbidity at different ages is important in the development of future interventions in primary care.

In total, 40 conditions were selected as outlined in detail previously.^15^ Definitions were based on a combination of Quality and Outcomes Framework business rules,^28^ Read Codes, and prescription data.

Multimorbidity was defined as the presence of two or more of these 40 conditions in one patient (32 physical and eight mental). To characterise the prevalence and type of multimorbidity, patients were grouped as follows:

- two or more physical conditions but no mental health conditions (physical-only);
- two or more mental health conditions but no physical conditions (mental-only); or
- two or more conditions including at least one physical and one mental (mixed physical and mental).

The top 10 most common conditions in those with multimorbidity were then examined in each age group. Conditions that shared a common vascular aetiology and usually have similar chronic management and treatment goals were classified as concordant (coronary heart disease [CHD], chronic kidney disease [CKD], diabetes, hypertension, heart failure, stroke/TIA, atrial fibrillation and peripheral vascular disease [PVD]), with the remainder being classified as discordant.^29^

As the current analysis focused on adults with multimorbidity, patients were divided into six age groups (25–34, 35–44, 45–54, 55–64, 65–74, and ≥75 years) to reflect different stages in the life course. Those <25 years were excluded as only 1.9% have multimorbidity.^15^

Associations between prevalence and socioeconomic deprivation were assessed using Spearman rank correlations. Ten conditions were also compared that featured in the top five ranking conditions across any age groups for the least and most deprived deciles. The *t*-test and one-way ANOVA were used to analyse differences in type of multimorbidity and prevalence of individual conditions between age groups and least and most deprived deciles. All analysis was conducted using Stata (version 11.1).

## RESULTS

### Multimorbidity prevalence

Table 1 shows differences in prevalence of morbidity in adults across the different age groups. The number and percentage of people with multimorbidity were higher in each successive age group, rising from 8.1% of 25–34-year-olds to 76.1% of those aged ≥75 years. By the age of ≥55 years, there were more people with multimorbidity than there were with a single condition or no condition.

**Table 1:** Prevalence of chronic morbidity

**Age group,** years	**Adult population (*n* = 1 272 685) *n* (%, 95% CI)**	**No conditions (*n* = 597 363) *n* (%, 95% CI)**	**One condition (*n* = 279 015) *n* (%, 95% CI)**	**Two or more conditions (*n* = 396 307) *n* (%, 95% CI)**
25–34	229 396 (18.0)	169 747 (74.0)	40 962 (17.9)	18 687 (8.1)
	(17.9 to 18.1)	(73.8 to 74.0)	(17.7 to 18.0)	(8.0 to 8.2)
35–44	278 993 (21.9)	179 338 (64.3)	60 771 (21.8)	38 884 (13.9)
	(21.8 to 22.0)	(64.2 to 64.4)	(21.7 to 21.9)	(13.8 to 14.0)
45–54	253 794 (19.9)	131 920 (52.0)	63 453 (25.0)	58 421 (23.0)
	(19.8 to 20.0)	(51.0 to 52.1)	(24.9 to 25.1)	(22.8 to 23.2)
55–64	219 333 (17.2)	75 843 (34.6)	58 171 (26.5)	85 319 (38.9)
	(17.1 to 17.3)	(34.5 to 34.7)	(26.3 to 26.7)	(38.7 to 39.1)
65–74	155 280 (12.2)	28 662 (18.5)	35 068 (22.6)	91 550 (59.0)
	(12.1 to 12.3)	(18.3 to 18.7)	(22.4 to 22.8)	(58.7 to 59.2)
≥75	135 889 (10.7)	11 853 (8.7)	20 590 (15.2)	103 446 (76.1)
	(10.6 to 10.8)	(8.5 to 8.9)	(15.0 to 15.3)	(75.9 to 76.3)

Analysis based on 40 chronic conditions: 32 physical and eight mental. Percentages in ‘adult population’ column relate to distribution of age groups within the sample, whereas percentages in the conditions columns relate to distribution within each separate age group.

Table 2 shows the prevalence of different types of multimorbidity (physical or mental or both) across age groups. Physical-only multimorbidity accounted for 56% of all multimorbidity overall, and was the most common type of multimorbidity from ≥55 years. In contrast, the number of people with mental-only multimorbidity accounted for <4% of multimorbidity overall, and this was most common below the age of 45 years. Mixed physical and mental multimorbidity accounted for almost 40% of all multimorbidity. It was the most common type of multimorbidity in all age groups <55 years, and continued to rise in prevalence after this.

**Table 2:** Type of multimorbidity for people with any multimorbidity

**Age group**	**Physical-only multimorbidity *n* (%, 95% CI)**	**Mental-only multimorbidity *n* (%, 95% CI)**	**Mixed physical and mental multimorbidity *n* (%, 95% CI)**
25–34 (*n* = 18 687)	5110 (27.3)	4019 (21.5)	9558 (51.1)
	(26.7 to 28.0)	(20.9 to 22.1)	(50.4 to 51.9)
35–44 (*n* = 38 884)	13 240 (34.0)	4987 (12.8)	20 657 (53.1)
	(33.5 to 34.5)	(12.4 to 13.2)	(52.6 to 53.6)
45–54 (*n* = 58 421)	26 642 (45.6)	3138 (5.4)	28 641 (49.0)
	(45.1 to 46.0)	(5.1 to 5.5)	(48.6 to 49.4)
55–64 (*n* = 85 319)	49 771 (58.3)	1626 (1.9)	33 922 (39.8)
	(58.0 to 58.6)	(1.8 to 2.0)	(39.4 to 40.1)
65–74 (*n* = 91 550)	62 536 (68.3)	665 (0.7)	28 349 (31.0)
	(68.0 to 68.6)	(0.6 to 0.8)	(30.7 to 31.3)
≥75 (*n* = 103 446)	65 912 (63.7)	586 (0.6)	36 948 (35.7)
	(63.4 to 67.0)	(0.5 to 0.7)	(35.4 to 36.0)
Total	223 211 (56.3)	15 021 (3.8)	158 075 (39.9)
	(56.1 to 56.4)	(3.7 to 3.9)	(39.7 to 40.0)

Analysis based on 40 chronic conditions: 32 physical and eight mental. Percentages are the % in each age group.

### Effect of deprivation on type of multimorbidity

Table 3 shows the prevalence of different types of multimorbidity (physical or mental or both) across age groups for the least and most deprived deciles. Physical-only multimorbidity had a similar prevalence in the most and least deprived deciles. In contrast, the number of people with mental-only multimorbidity was markedly higher in the most deprived than in the least deprived especially in the younger age groups. The prevalence was similar in both deprivation groups ≥65 years. Mixed physical and mental multimorbidity was two- to threefold more common in the most deprived compared with the least deprived in all age groups <75 years.

**Table 3:** Differences between types of multimorbidity by age group and deprivation

**Age group, years**	**Physical-only multimorbidity *n* (%, 95% CI)**		**Mental-only multimorbidity *n* (%, 95% CI)**		**Mixed physical and mental multimorbidity *n* (%, 95% CI)**	
	**Most deprived**	**Least deprived**	**Most deprived**	**Least deprived**	**Most deprived**	**Least deprived**
25–34	447 (1.9)	340 (1.9)	613 (2.6)	159 (0.9)	1289 (5.4)	465 (2.7)
	(1.7 to 2.1)	(1.7 to 2.1)	(2.4 to 2.8)	(0.7 to 1.1)	(5.1 to 5.6)	(2.4 to 2.9)
35–44	1061 (4.3)	1040 (4.1)	815 (3.3)	231 (0.9)	2844 (11.5)	1006 (3.9)
	(4.1 to 4.5)	(3.8 to 4.3)	(3.0 to 3.5)	(0.7 to 1.1)	(11.1 to 11.9)	(3.7 to 4.1)
45–54	2087 (10.3)	2279 (8.9)	440 (2.2)	184 (0.7)	3795 (18.8)	1567 (6.1)
	(10.0 to 10.7)	(8.5 to 9.2)	(2.0 to 2.4)	(6.2 to 8.3)	(18.2 to 19.3)	(5.8 to 6.4)
55–64	3301 (22.9)	4556 (20.4)	147 (1.0)	123 (0.6)	3705 (25.7)	2156 (9.7)
	(22.1 to 23.8)	(19.9 to 20.9)	(0.8 to 1.2)	(4.5 to 6.5)	(24.9 to 26.4)	(9.2 to 10.0)
65–74	4423 (39.1)	5316 (37.9)	50 (0.4)	65 (0.5)	3128 (27.6)	1902 (13.6)
	(38.2 to 39.9)	(37.1 to 38.7)	(0.2 to 0.4)	(0.3 to 0.5)	(26.8 to 28.4)	(13.0 to 14.1)
≥75	4177(46.1)	6200 (48.1)	37 (0.4)	52 (0.4)	2887 (31.8)	3488 (27.0)
	(45.0 to 47.1)	(47.2 to 48.9)	(0.2 to 0.5)	(0.3 to 0.5)	(30.8 to 32.8)	(26.2 to 27.8)

Analysis based on 40 chronic conditions: 32 physical and eight mental. Percentages are the % in each age group/deprivation group (n = 103 695 most deprived, 117 708 least deprived).

**Figure 1:**
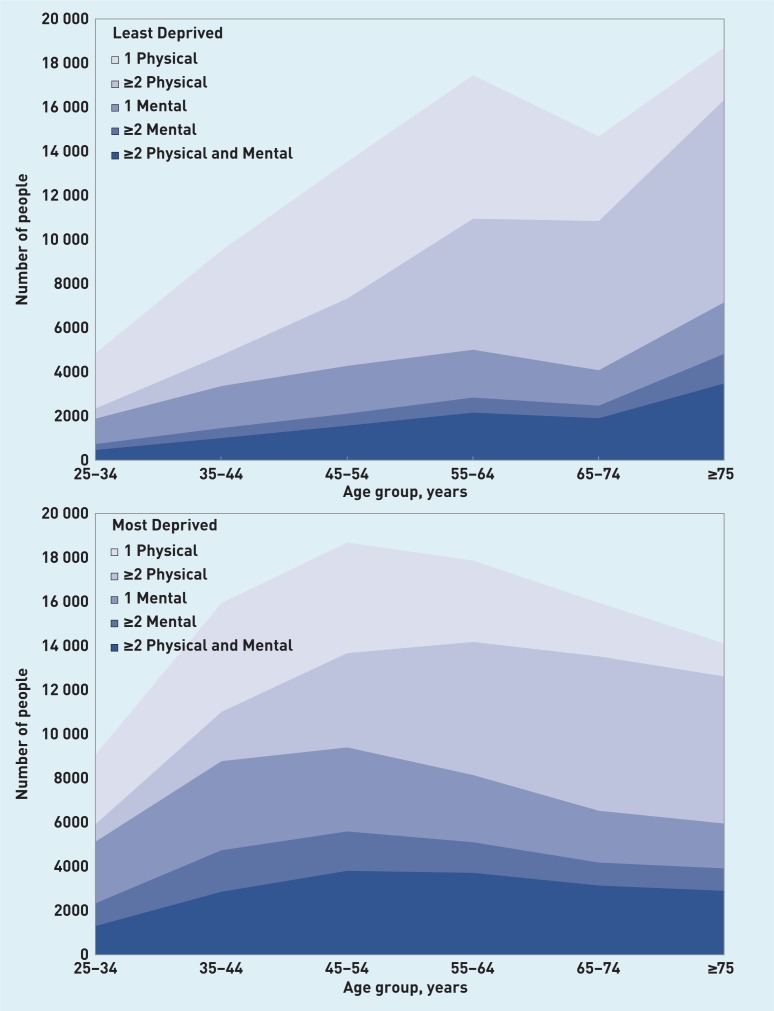
***Number and type of chronic condition for overall population by number of patients (least and most deprived deciles).***

Figure 1 shows the numbers of people with the different types of multimorbidity by age group, in the most and least deprived deciles, and, for comparison, also shows the numbers with no condition or one condition (mental or physical). Although multimorbidity is more common in older people, the shape of the population distribution means that the absolute numbers of people with multimorbidity vary less by age, and there are particularly large numbers of people in the most deprived decile with mental health problems (multiple or mixed).

### Prevalence of individual conditions in multimorbid patients

Table 4 shows the top 10 most prevalent conditions in multimorbid patients in each age group. Depression was the most prevalent condition for multimorbid patients in all age groups <55 years, whereas hypertension was the most prevalent condition for those aged ≥55 years. Depression and pain featured in the top five conditions across all age groups.

**Table 4:** Age differences between the top 10 most prevalent conditions in multimorbid patients

**Rank order of conditions**	**Age 25–34 % (95% CI) *n* = 18 687**	**Age 35–44 % (95% CI) *n* = 38 884**	**Age 45–54 % (95% CI) *n* = 58 421**	**Age 55–64 % (95% CI) *n* = 85 319**	**Age 65–74 % (95% CI) *n* = 91 550**	**Age ≥75 % (95% CI) *n* = 103 446**
**1**	Depression 46.1 (45.3 to 46.8)	Depression 46.9 (46.4 to 47.4)	Depression 38.8 (38.4 to 39.2)	Hypertension 48.5 (49.2 to 48.9)	Hypertension 58.3 (58.0 to 58.6)	Hypertension 61.9 (61.5 to 62.3)
**2**	Drug misuse 25.9 (25.2 to 26.5)	Pain 26.4 (25.9 to 26.8)	Hypertension 30.9 (30.4 to 31.2)	Pain 31.3 (31.0 to 31.6)	Pain 30.0 (29.7 to 30.3)	CHD 31.2 (30.9 to 31.5)
**3**	Asthma 23.1 (22.4 to 23.7)	Asthma 19.0 (18.6 to 19.4)	Pain 29.6 (29.2 to 30.0)	Depression 28.0 (27.8 to 28.3)	CHD 26.1 (25.8 to 26.4)	Pain 23.6 (23.3 to 23.8)
**4**	Anxiety 19.8 (19.3 to 20.4)	Anxiety 17.8 (17.4 to 18.1)	Dyspepsia 18.4 (18.1 to 18.7)	Diabetes 17.7 (17.4 to 18.0)	Diabetes 21.1 (20.8 to 21.4)	CKD 18.5 (18.2 to 18.7)
**5**	Pain 19.1 (18.6 to 19.7)	Dyspepsia 16.5 (16.1 to 16.8)	Asthma 14.2 (13.9 to 14.4)	Dyspepsia 17.2 (16.9 to 17.4)	Depression 18.5 (18.3 to 18.8)	Depression 17.2 (17.0 to 17.4)
**6**	Alcohol dependence 14.5 (14.0 to 15.0)	IBS 15.2 (14.8 to 15.5)	Diabetes 13.6 (13.4 to 13.9)	CHD 15.9 (15.6 to 16.1)	Dyspepsia 15.9 (15.7 to 16.2)	Diabetes 17.2 (17.0 to 17.5)
**7**	IBS 14.4 (13.9 to 14.9)	Drug misuse 14.8 (14.4 to 15.1)	Anxiety 13.6 (13.3 to 13.9)	Thyroid 13.9 (13.7 to 14.2)	COPD 14.6 (14.4 to 14.8)	Constipation 17.0 (16.7 to 17.2)
**8**	Dyspepsia 10.4 (10.0 to 10.8)	Hypertension 13.7 (13.3 to 14.0)	IBS 13.5 (13.3 to 13.8)	IPA 13.3 (13.1 to 13.7)	Thyroid 14.5 (14.3 to 14.8)	Stroke 16.6 (16.4 to 16.8)
**9**	Thyroid 7.5 (7.1 to 7.8)	Alcohol dependence 13.3 (13.0 to 13.6)	Thyroid 13.1 (12.8 to 13.4)	COPD 11.1 (10.8 to 11.3)	IPA 13.7 (13.5 to 13.9)	Thyroid 15.9 (15.7 to 16.1)
**10**	Hearing loss 6.9 (6.6 to 7.3)	Thyroid 10.9 (10.6 to 11.2)	Alcohol dependence 12.3 (12.0 to 12.6)	Anxiety 10.8 (10.6 to 11.0)	Stroke 10.5 (10.3 to 10.7)	Hearing loss 15.5 (15.3 to 15.7)

CKD = chronic kidney disease. CHD = coronary heart disease. COPD = chronic obstructive pulmonary disease. IBS = irritable bowel syndrome. IPA = Inflammatory poly-arthropathy and includes rheumatoid arthritis and related conditions, systemic connective tissue disorders, and gout. Drug misuse includes prescription drug misuse of varying degrees.

Ten conditions made up the top five most common conditions in multimorbid patients in each age group: seven physical conditions (four ‘concordant’ conditions with related aetiology and/or management: diabetes, CHD, hypertension, and CKD; and three ‘discordant’: pain, asthma, and dyspepsia), and three mental health conditions (depression, anxiety, and drugs misuse, which includes use of prescription drugs that GPs have coded as being problematic in some way). The prevalence of concordant physical conditions in multimorbid patients increased with age, whereas in younger age groups multimorbidity was characterised by discordant physical conditions and mental health conditions.

### Effect of deprivation on the prevalence of individual conditions in multimorbid patients

**Figure 2:**
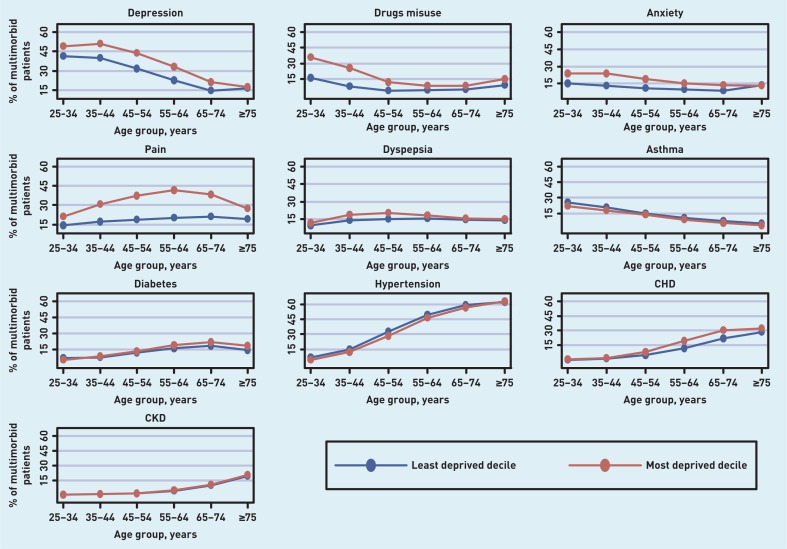
***Prevalence of most common conditions in multimorbid patients, by age and deprivation. CKD = chronic kidney disease. CHD = coronary heart disease***

The prevalence of the ten conditions that feature in the top five conditions across age group is shown in Figure 2, by age and deprivation (most and least deprived deciles). Deprivation was associated with a higher prevalence of seven out of the 10 conditions (depression, drugs misuse, anxiety, dyspepsia, pain, CHD, and diabetes). Deprivation correlated with drug misuse and pain across all age groups; in depression and anxiety in all age groups up to those aged ≥75 years; in dyspepsia in all age groups <65 years; in CHD for those aged ≥45 years; and in diabetes for those ≥55 years.

## DISCUSSION

### Summary

Multimorbidity was characterised by age and deprivation in a large nationally representative sample. The prevalence of multimorbidity increased with age, with physical-only multimorbidity being the most common pattern in the over-55s, and involved many ‘concordant’ conditions.^30^ In earlier adulthood, multiple mental health conditions and mixed physical and mental conditions were more prevalent. Mixed physical and mental multimorbidity was much more common in the deprived compared with the affluent at all ages <75 years. Depression and pain featured in the top five conditions across all age groups, and 10 conditions (seven physical and three mental) accounted for the top five most common conditions in all age groups. Deprivation was associated with a higher prevalence of seven of these 10 conditions.

The higher prevalence of mixed mental and physical conditions that exists in the more deprived, particularly at an earlier age, may reflect previous evidence, which shows that mental health conditions are more prevalent in people with increasing physical disorders.^30^ It was not possible, however, to assess which condition came first for those with multimorbidity. Therefore, it may be that the higher prevalence of physical conditions such as CHD, diabetes, and pain found in the most deprived is influenced by higher rates of mental conditions occurring first, at an earlier age. Much higher rates of alcohol dependence and drugs misuse were found in the more deprived across all age groups, but particularly in those <45 years of age. This, along with higher rates of smoking, which are often found in the more deprived, could be a contributing factor to the development of additional physical conditions, but also other mental conditions such as depression and anxiety.^31^ Further work is required to assess which conditions come first and whether these differ by age and deprivation.

### Strengths and limitations

There are several limitations of this study. First, as it is a secondary data analysis the study is reliant on the quality of primary data recording. Some of the conditions included in this study are likely to be under-recorded, implying that any findings will underestimate the true prevalence of multimorbidity. Second, multimorbidity is defined using a simple count of conditions, which does not take account of the varying effect on individuals of different combinations of conditions or their severity. Third, the way that different ‘conditions’ are defined in the database may lead to some problems. For instance, the relatively high prevalence of hearing loss in the youngest age group may be explained by its definition as ‘ever recorded’. This could, therefore, include glue ear at a younger age. The trade-off in this case is that if hearing loss had been defined as ‘recorded in the last 5 years’, it would be liable to undercount persistent deafness in older people, which is already likely to be under-recorded. Similarly, using prescribing as part of the definition of certain conditions (such as anxiety) has the potential both to underestimate (for example, in the case of anxiety that is managed non-pharmacologically) and overestimate (for example, when anxiolytics are used for other conditions) the true prevalence of those conditions. Such problems are common to all similar database studies and this study’s approach has been to be pragmatic and transparent, with full condition definitions published.^15^

A strength of the study is its large size and the representativeness of the Scottish nation. It includes 40 conditions, which is substantially more than most similar studies.^12^ It also covers all age groups and is representative of the whole population in terms of age, sex, and socioeconomic status, making the findings more broadly applicable to other settings than most similar studies.^21^,^23^,^32^,^33^

### Comparison with existing literature

It is believed that this is the largest study to examine multimorbidity by age and deprivation using a wide range of conditions. A study in Canada of the presence of 16 conditions in 5010 adults aged >18 years found that age, sex, income, and family structure were independently associated with multimorbidity.^20^ Much of the focus of multimorbidity research has been on older age groups, however, with little assessment of the impact of socioeconomic deprivation.^34^ Results from this study are in line with previous findings showing that although multimorbidity increases with age, the absolute numbers of people with multimorbidity are more evenly spread across ages than generally assumed, particularly in socioeconomically deprived populations.^23^,^31^,^32^,^35^ Studies that have examined multimorbidity across age groups have generally focused on overall multimorbidity count rather than on the prevalence of individual conditions in the multimorbid.^15^,^21^ An Australian study of 1651 patients with multimorbidity (using a smaller number of conditions than in the present study) found that adults <40 years of age most commonly had a mental health condition (anxiety, depression, stress-related problem, other mental health problem) and asthma as a cluster, whereas in those aged 40–59 years the most common cluster was a mental health condition and arthritis.

Higher rates of multimorbidity and particularly mental health conditions in more deprived areas have been found elsewhere.^34^,^36^–^37^ The higher prevalence of depression and pain in more deprived areas found in this analysis contrasts with results from a German study of 3189 multimorbid people aged ≥65 years, which found an association between higher rates of multimorbidity (based on 46 chronic conditions) and deprivation, but no association in the older people studied between deprivation and the presence of one or more conditions in a study defined cluster of ‘anxiety, depression, somatoform disorders, and pain’.^38^ A cross-sectional analysis of 7305 participants aged ≥50 years in the US investigated the association between childhood financial hardship, lifetime earnings, and multimorbidity.^39^ The study found that childhood financial hardship and lifetime earnings are associated with multimorbidity, but not associated with the absence of morbidity.^38^

### Implications for research and practice

Results from this study highlight key challenges for organisation and delivery of integrated health and social care, especially for adults <65 years of age (most people with multimorbidity) and particularly in deprived areas. Although a relatively small proportion of the population of younger adults (<45 years) had multimorbidity, the mix of mental health, pain, and substance misuse in early adult life may increase premature mortality and/or the potential for this group of young adults (if they survive) to develop ‘high burden’ multimorbidity in later life.^11^,^25^,^40^,^41^

The higher prevalence in more deprived areas of mixed physical and mental multimorbidity highlights the need for holistic and integrated primary care services led by generalists, which if not met — because of the persistence of the inverse care law — is likely to widen inequalities in health.^42^–^44^ This threat is potentially greatest for the under 65s with multimorbidity, as geriatricians often work in a way that complements and supports generalist GPs, whereas in many countries there are fewer, if any, secondary care generalist physicians for younger adults. Existing approaches to medical education, research, and healthcare delivery focus on single diseases, but a growing body of research has called for a redesign in health systems to meet the challenges of increasing multimorbidity.^15^ Evidence has shown that physical multimorbidity is strongly associated with unplanned admission to hospital, including admissions that were potentially preventable.^39^ Furthermore, the risk of admission to hospital was exacerbated by the coexistence of mental health conditions and socioeconomic deprivation and was also higher in the youngest age groups compared with the middle-aged. It has also been shown that for those with mental health problems there is a marked social patterning for hospital admissions.^45^ Given the higher rates of combined physical and mental multimorbidity in the younger age groups, particularly in the most deprived areas, this suggests that focusing on these people could offer potential for a reduction in preventable admissions. The evidence base for the effect of multimorbidity on mortality and hospital admissions remains small, however, and further work is required in this area.

Results from this study add weight to this call but also suggest the need for interventions, particularly in the most deprived areas, aimed at the younger adults and the large numbers of patients with coexisting physical and mental multimorbidity.

Mixed physical and mental multimorbidity is common across the life-span and is exacerbated by deprivation from early adulthood onwards. Individuals in more deprived areas face the challenge of multimorbidity in greater numbers characterised by mixed mental and physical conditions from an earlier age and lasting longer over the life-course. The findings of this study highlight the need for longitudinal studies that can take a life-course approach to aetiological understandings of the determinants of different patterns of multimorbidity and how they interact over the life-span.
